# Reply: Is CD1a involved in antitumour immune responses during carcinogenesis?

**DOI:** 10.1038/sj.bjc.6601598

**Published:** 2004-02-17

**Authors:** B J Coventry, S Heinzel

**Affiliations:** 1 Tumour Immunology Laboratory, Department of Surgery, University of Adelaide, Royal Adelaide Hospital, North Terrace, Adelaide 5000, South Australia

**Sir**,

We agree with Dr Cappello's comment relating to our paper^1^ that the CD1a expression by dendritic cells (DC) in human cancers is a very interesting observation and that the possible role of the CD1a molecule in the presentation of tumour-associated antigens requires further investigation. We were intrigued to note in the Cappello *et al* paper^2^ that CD1a was expressed in 75–94% of epithelial biopsy tissue sections from patients with Barrett's metaplasia of the oesophagus, but that this was essentially not found in patients with dysplasia or neoplasia, nor in normal gastric or colonic tissues examined. Although the study was small, the report brief, and did not contain more advanced invasive carcinomas, it might indicate that transitory expression of CD1a by epithelia may occur during the initial phases of carcinogenesis. We have some unsubstantiated data that also indicate that some *in situ* breast carcinomas may also show expression of CD1a. It is unclear whether the expression of the CD1a molecules is actually on the epithelial cells *per se* or on intraepithelial DC. We have theorised that if CD1a expression occurs on both epithelial cells and DC, then this may indicate a similar arrangement to that observed for MHC molecular expression for presentation of peptide antigens. This would strengthen our hypothesis that CD1a, with some known structural similarity to MHC, may be very important for the presentation of tumour-derived glycolipid antigens to T cells. In addition, we are currently investigating CD1a restricted T-cell responses against tumour antigens, and we hypothesise that these are important for effective antitumour immune responses *in vivo*. If epithelial cells in transition towards neoplasia express CD1a together with neo-antigens, this may be a sufficient ‘danger’ signal to alert the immune system to early carcinogenesis, thus inducing an effective immune response in some individuals – perhaps as a mechanism for epithelial protection against further malignant progression. The failure of expression of CD1a by epithelia in transition may not allow an immune response to occur, perhaps explaining the worse prognosis of CD1a-negative oesophageal lesions noted in the study of Cappello *et al*. This theory remains to be (dis)proved.
